# The Prevalence of Helicobacter Pylori babA, homB, aspA, and sabA Genes and Its Relationship with Clinical Outcomes in Turkey

**DOI:** 10.1155/2019/1271872

**Published:** 2019-06-13

**Authors:** Nimet Yılmaz, Meltem Koruk Özer

**Affiliations:** ^1^University of SANKO, Faculty of Medicine, Department of Internal Medicine, Division of Gastroenterology, 27310 Sehitkamil, Gaziantep, Turkey; ^2^Medical Biology and Genetics Department, Health Sciences İnstitute, University of Gaziantep, Turkey

## Abstract

**Background and Aims:**

The cag A and vac A genes of* Helicobacter pylori (H. pylori) *are closely associated with the pathogenicity of bacteria. However, the significance of H. pylori* babA, homB, aspA*, and* sabA *genes is not clear in phenotypic characteristics of virulence. This study aimed to investigate the frequency and importance of these genes in patients with H. pylori positive peptic ulcer (PU).

**Materials and Methods:**

Patients with a PU or nonulcer dyspepsia (NUD) based on the upper gastrointestinal (UGI) endoscopy findings were included in the study. Biopsy samples from antrum and corpus were cultured into Columbia agar.* H pylori* were characterized by urease, catalase, oxidase test, and gram staining. Genomic DNA was extracted and stored. The* babA, homB, aspA*, and* sabA* genes were determined by using polymerase chain reaction analysis.

**Results:**

A total 214 patients were included (99 PU and 115 NUD) and H. pylori could be isolated in 82 patients (36 PU and 46 NUD). The frequency of the* babA* (25% vs. 15.2%, p=0.25),* homB* (2.7% vs. 4.3%, p=1),* aspA* (69.4% vs. 73.9%, p=0.2), and* sabA* (2.7% vs. 10.8%, p=0.88) genotypes was not different between PU and NUD patients. There were some correlations between the presences of these genes.

**Conclusion:**

This study managed to determine* babA, homB, aspA, *and* sabA genes of *H. pylori by PCR. However, the frequency of these factors was not different in patients with PU and NUD. There is no role of* babA, homB, aspA*, and* sabA genes *for the development of peptic ulcer in Turkish population.

## 1. Introduction

Helicobacter pylori (*H. pylori*) is a gram-negative, spiral-shaped, 4-6-flagellated mobile bacterium that grows in the digestive tract and microaerophilic environment at 37°C in culture. The coccoid form of* H. pylori* is also called sleeping form*. H. pylori* produces urease that catalyzes the hydrolysis of urea to yield ammonia and carbonic acid. Flagella, urease, and adhesins are all essential factors for* H. pylori* to colonize the gastric mucosa [1].

*H. pylori* may be present in almost half of the world population. The incidence of* H. pylori* infection varies according to gender, race, and social and socioeconomic status of the population. The people who are living in developing countries are very commonly infected with* H. pylori* whereas the frequency of H. pylori infection is rare in Australia, Canada, and the USA [2]. The occurrences of new gastric cancer cases were variable in developing countries (8.4%) and developed countries (4.5%) [3]. Gastrointestinal cancer-related death rate is the third most common cause of all cancer-related deaths.* H. pylori* are correlated with the development of duodenal ulcer and gastric cancer.* H. pylori *infected individuals are having risk of developing peptic ulcer in 15-20%, gastric cancer in 1%, and primary gastric lymphoma in 0.1%.* H. pylori* infection is a high risk factor for the development of peptic ulcer, gastric maltoma, and adenocarcinoma [4]. Therefore, it may cause significant health problems. The transmission way of* H. pylori* has not been fully clear, yet [5].

*H. pylori* are adapted and colonize harsh, acidic environment of the stomach and survive in acidic environment that causes induction of gastritis, peptic ulcer, or gastric cancer.* H. pylori* is actually an opportunistic pathogen. Some virulence factors of* H. pylori,* such as* cagA* and* vacA*, are the most pathogenic factors among all virulence factors [6]. There are also some other genes of* H. pylori* such as* babA, homB, aspA*, and* sabA *the significance for pathogenicity of which is not clear yet.

*H. pylori* adhesions such as the Lewis blood group antigen-binding adhesion (*babA*) and the sialic acid-binding adhesion (*sabA*) are considered to have a significant function on initial colonization of* H. pylori* [7, 8].

*H. pylori* outer-membrane proteins (*hom*) family is a small protein family including the C-terminal hydrophobic motif and signal sequences of outer membrane proteins. The* hom* family is one of the outer-membranes coding gene family that is divided into two families:* homA* and* homB* which are 90% identical; the difference is related to central domain [9].

Recent studies on adherence features of* H. pylori* have reported that* babA* promotes attachment of* H. pylori* to the gastric epithelial cells. The* babA* facilitates entry of* cagA* and* vacA* virulence factors into host cells [10, 11].

The second adhesion is* sabA* first identified in the* babA*-mutant* H. pylori *strain [8]. The* sabA* binds to sialylated carbohydrates on the surface of neutrophils. From this perspective,* sabA *is thought to promote immune response [11].

The aim of this study was to determine* H. pylori babA, homB, aspA*, and* sabA* genes and to identify the rate of these virulence genes in the biopsy samples by PCR analysis.

## 2. Materials and Methods

### 2.1. Collection of Biopsy Samples

A total of 214 patients were included in this study: 115 nonulcer dyspepsia and 99 peptic ulcer. The patients were from south east part of Turkey undergoing upper gastrointestinal endoscopy at the endoscopy unit of the Department of Gastroenterology, University of Gaziantep. During endoscopy, biopsy samples were taken and the obtained tissues were placed into 0.8% serum physiologic solution and then cultured immediately. Informed consent was taken from all patients and The Ethics Committee of Medical School of University of Gaziantep approved the study. Results were confirmed both clinically and microbiologically.

### 2.2. Microbiologic Analysis

#### 2.2.1. Culturing

In order to prevent contamination, aseptic conditions were provided. The obtained tissues were immediately placed into a liquid 0.8% serum physiologic solution and inoculated into Columbia agar with 5% sheep blood (BD, Heidelberg, Germany), containing* H. pylori* selective supplement (OXOID LTD, Basingstoke, Hampshire, England) to eliminate another bacterial contamination, and then incubated under anaerobic conditions, 5% CO_2_ at 37°C for 4-6 days.

#### 2.2.2. Urease, Catalase, and Oxidase Tests

To prove existence of* H. pylori*, catalase (Merck, Darmstadt, Germany), urease (Merck, Darmstadt, Germany), and oxidase (Merck, Darmstadt, Germany) tests were performed and also* H. pylori* morphology was identified.

#### 2.2.3. Gram-Staining

To observe* H. pylori* under the light microscope, gram staining method was performed. Crystal violet (Merck, Darmstadt, Germany) was applied to heat-fixed smear of bacterial culture. Lugol (Merck, Darmstadt, Germany) that binds crystal violet was added. To decolorize it, ethanol (Merck, Darmstadt, Germany) was added and then stained with safranin (Merck, Darmstadt, Germany).

### 2.3. Genotyping of* H. pylori*

#### 2.3.1. DNA Isolation

Genomic DNA was extracted from histopathologically confirmed cases of nonulcer dyspepsia and peptic ulcer using Qiagen DNA isolation kit Qiagen, QIAmp DNA Mini Kit (Hilden, Germany) according to manufacturer's instructions. The DNA was stored at -20°C until used for molecular studies.

#### 2.3.2. PCR Analysis

Touchdown PCR protocols were performed using Dream Taq DNA Polymerase (Thermo Scientific, Lithuania, EU) kit. PCR amplifications were performed on 50 *μ*l master mixture that contained 100 ng of genomic DNA, 10 pmole each of primers, 10X buffer, 2 mM each of nucleotides (Deoxynucleotide Triphosphate, Thermo Scientific, Lithuania, EU), and 0.5 units of Taq Polymerase. PCR annealing temperatures for primers of aspA, babA, cagA, homB, sabA, and vacA were 59°C, 55°C, 58°C, 58°C, 56°C, and 55°C, respectively. PCR products were then electrophoresed for 45 min at 130 Volt on 1% agarose gel in the presence of 0.5g/mL of ethidium bromide (Sigma, Steinheim, Germany) and illuminated under UV light (UVP EC3 imaging system, Upland, CA, USA).

#### 2.3.3. Statistical Analysis

Comparisons of variables were performed with the chi-square test, One-way ANOVA test, and Tukey's Multiple Comparison Test (GraphPad Prism 5) to compare the differences among nonulcer dyspepsia and peptic ulcer patients.* p* values <0.05 were considered significant.

## 3. Results

**Figure 1:**
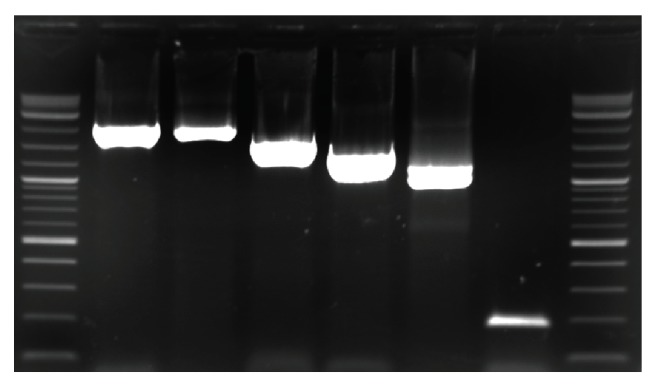
*Characterization of virulence factors of H. pylori by PCR*. Each lane shows different virulence factors of* H. pylori* strain isolated from human samples. M: Marker, A: babA (2192 bp), B: cagA (1741 bp), C: VacA (1624 bp), D: AspA (1401 bp), E: HomB (1005 bp), and F: sabA (187 bp).

Gastric biopsies from all patients included in the study were cultured and initially assessed for the presence of* H. pylori *by urease, catalase, and oxidase tests. As a result of these tests,* H. pylori *were detected in 82 patients (38.32%), whereas bacteria could not be detected in 132 patients (61.68%). All* H. pylori*-positive patients (82) were further analyzed for the presence of* H. pylori* virulence factors by PCR using* babA, homB, aspA*, and* sabA-*specific primers encoding (Table 1(a))* babA, homB, aspA*, and* sabA *genes (Figure 1).

A total of 82* H. pylori*-positive patients (46 nonulcer dyspepsia (25 females, 21 males) and 36 peptic ulcer (16 females, 20 males)) were enrolled in this study (Table 1(b)). The mean age of the overall population was 45.7±16.5 years. There were significant relationships between gender and the nonulcer dyspepsia and peptic ulcer diseases related to these virulence factors (*p*<0.001). However, there were no significant differences in mean age (*p*>0.05).

### 3.1. Virulence Factors

**Figure 2:**
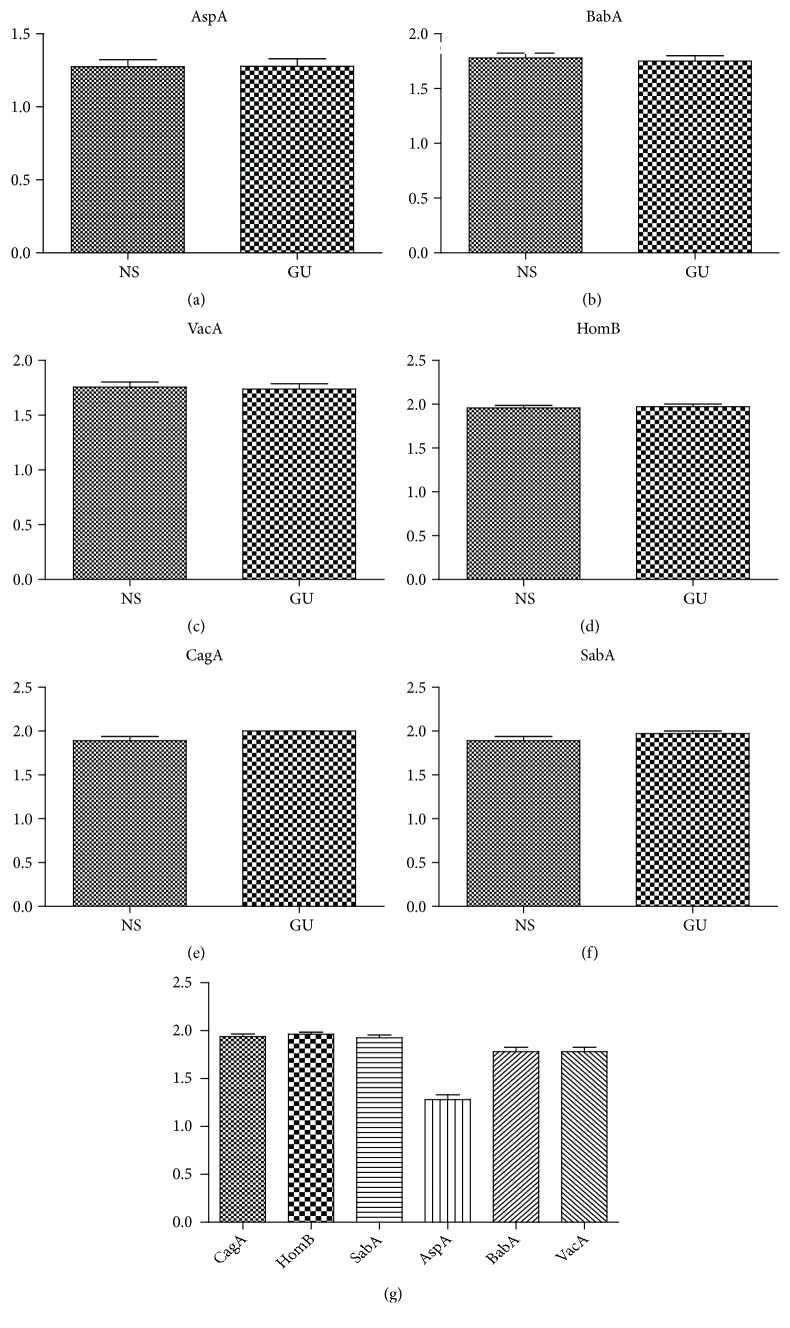
PCR application of the biopsy samples taken from H. pylori positive patients with nonulcer and gastric ulcer samples. The presence of AspA (a), BabA (b), VacA (c), HomB (d), CagA (e), and SabA (f) genes in nonulcer and gastric ulcer samples by using PCR. (g) The comparison of* H. pylori* virulence factors in normal and gastric ulcer samples.

*(i) Blood Group Antigen-Binding Adhesin, babA*. The* babA* gene of* H. pylori* was determined in 16 patients (19.51%), whereas 66 patients (80.49%) were classified as* babA*-negative (Table 1(c)). Out of 16* babA* gene positive patients, 7 of them (43.75%) were from nonulcer dyspepsia patients and 9 of them (56.25%) were from peptic ulcer patients (Table 1(b)). The presence of* babA* was statistically significant in nonulcer dyspepsia (*p *< 0.001) and peptic ulcer (*p *< 0.001) (Table 2(b)) (Figures 2(a)–2(g)).

*(ii) Helicobacter Outer Membrane Family Member, homB*. The 1005-bp PCR product indicating the presence of* homB* gene was detected in 3 patients (3.66%), whereas 79 patients (96.34%) were negative for* homB* gene (Table 2(a)). Out of 3* homB*-positive strains, 2 isolates (66.6%) were from nonulcer dyspepsia patient and 1 isolate (33.3%) was from patient diagnosed with peptic ulcer disease. The presence of* homB* was associated with the presence of* aspA* (*p* <0.001) and* babA* (*p* <0.05) (Table 2(a)). A statistically significant correlation between* homB* and* aspA* and* babA* gene was detected. Moreover, the status of* homB *had significant effect on nonulcer dyspepsia (*p *< 0.001) and peptic ulcer patients (*p *< 0.001) (Figure 2(d)) (Figures 2(a)–2(g)).

*(iii) Aspartate Ammonia-Lyase, aspA*. Fifty-nine biopsies that were obtained from different patients (71.94%) were positive for the* aspA* gene, with the remaining 23 (28.04%) being* aspA*-negative as a result of the 1401-bp PCR product (Table 1(c)). Out of 59* aspA*-positive strains, 34 isolates (57.62%) were from nonulcer dyspepsia patients, and 25 isolates (42.38%) were from patients diagnosed with peptic ulcer disease. The frequency of* aspA *(71.95%) (*p*<0.0001) was significantly higher compared to the frequency of* babA* (19.51%),* homB* (3.65%), and* sabA* (7.31%) in nonulcer dyspepsia and peptic ulcer patients. The presence of* aspA* was associated with the presence of* babA, homB*, and* sabA *(*p *< 0.0001) (Table 2(a)). There was a positive correlation between* aspA* and* babA, homB, sabA*, and* vacA *genes. The presence of* aspA* had statistically significant impact on nonulcer dyspepsia (*p *< 0.001) and peptic ulcer (*p *< 0.001) (Table 2(b)) (Figures 2(a)–2(g)).

*(iv) Sialic Acid-Binding Adhesin, SabA*. The 187-bp PCR product indicating the presence of* sabA* gene of* H. pylori* was determined in 6 patients (7.31%), whereas 76 patients (92.69%) were classified as* sabA*-negative (Table 1(c)). Out of 6* sabA*-positive patients, 5 of them (83.33%) were from nonulcer dyspepsia patients and 1 of them (16.66%) was from peptic ulcer patient. The presence of* sabA* was just associated with the presence of* aspA* (p < 0.001). Furthermore, the presence of* sabA* gene had significant effect on nonulcer dyspepsia (*p *< 0.001) and peptic ulcer (*p *< 0.001) (Table 2(b)) (Figure 2(f)).

Possible combinations of all of these virulence factors were determined in Turkish population (Figures 2(a)–2(g)).

## 4. Discussion

*H. pylori *is a gram-negative bacillus which causes gastritis, peptic ulcer, and gastric cancer [12]. The prevalence of* H. pylori* depends on geographic regions, age, social and economic status, occupation, and living environment [6, 13].* H. pylori *have genetically diverse strains, and the strains differ in virulence [14].

In this study, the distribution of* aspA, babA, homB*, and* sabA *genes in* H. pylori* isolated from patients suffering from gastroduodenal diseases in Turkey determined using PCR analysis and the relationship between these virulence factors was assessed.

Studies have reported that there is a relationship between* babA*-positive* H. pylori *and gastric inflammation in humans. Furthermore, the* babA*-positive* H. pylori *increased risk of peptic ulcer and gastric cancer in humans [15, 16].

The* babA* gene has been detected on the outer membrane of the* H. pylori* strain. It has been shown that the* babA* is able to induce DNA double-strand breaks (DSBs) in the cells, but DSBs are the strictest type of DNA destruction and can cause chromosomal aberrations, such as deletions, insertions, and translocations resulting in loss of heterozygosity which are hallmarks of gastric cancer [17].

*H. pylori* strains that were isolated from East Asia expressed* babA *gene but* H. pylori* strains from 24 western countries did not express* babA* gene. These bacterial strains caused mild gastric problem. A meta-analysis review revealed that the existence of* babA* is correlated with high risk of peptic ulcer (OR = 2.069), especially the duodenal ulcer (OR = 1.588). This type of association was observed only in Western countries and not in Asian countries [18].

During the first 2-12 weeks during the experimental* H. pylori* infection, the* babA* expression disappeared in the experimental animals [19, 20].

*H. pylori *that was isolated from patient samples showed incredible variety at the* babA* locus, which can translate distinct adhesin that binds only blood group (O/Le^b^, or A/ALe^b^ and B/BLe^b^) [21].

We have also observed the same results as documented in literatures. The* babA* expression is a dynamic process. The vigorous and variety nature of host glycosylation adds extra complexity. It has been shown that loss of* babA* expression associated with gender. For this conclusion, the mice model has been used [22]. The* oorA, scoD, aroQ, fld A*, and* aspA* of* H. pylori* proteins are thought to hypothetically interact. It is deduced that these proteins also play a role in oxidation reduction [23].

Significant increase in protein with antioxidant activity (*aroQ, aspA, fldA, icd, OorA*, and* scoB*) and high acid environment adaptation proteins (*katA* and* napa*) in* H. pylori* has been shown to be high [23]. It has been shown that an increase in the expression of three genes encoding enzymes involved in intrabacterial ammonia production was observed. The genes are amidase* amiE* and* amiF *and* aspA*. These enzymes can help neutralize the protons entering under acidic environmental conditions by producing intrabacterial ammonia. This study revealed that aspA is taking part in intrabacterial ammonia production [24].

The cytotoxin-associated gene (*cagA*) and vacuolating cytotoxin (*vacA*) are* H. pylori* virulence factors and associated with gastric ulcer, gastric cancer [25, 26]. However, it has been documented that there is no difference between the presence of the homB gene and severe gastric diseases [27]. Besides, the disease reason has been correlated with many outer membrane proteins (OMPs). Particularly, the outer membrane proteins of H. pylori such as* alpA*,* alpB, babA, homB, hopZ, oipA, and sabA* are all correlated with variable disease outcomes [28]. H. pylori has a high content of simple sequence repeats, mainly in genes encoding outer membrane proteins [29]. The* cagA* and* vacA* are polymorphic genes. The outer membrane proteins families are strictly correlated paralogs. For instance, the bab- genes family consists of three paralogs* babA, babB*, and* babC*. These paralog* H. pylori* genes can be located at three different chromosomal loci [30].

During infection and an increase of* H. pylori *colonization,* homB*, outer membrane protein, is very crucial for adherence of the* H. pylori* to the gastric epithelium. A statistically significant correlation between* homB* and* aspA* (*p* < 0.0001) and* babA *(*p*< 0.05) gene was detected in this study. On the other hand, there was no significant relationship between* homB* and* sabA* gene according to this study [31].

The* hom* gene family is a small paralogous protein. The* hom*B and* hom*A genes are almost the same which are 90% [24]. The* hom*B was observed more often than* hom*A in East Asia. The* hom*B was correlated with an enlarged risk of peptic ulcer disease in East Asia. The* homB* has a role in proinflammation. The* homB* was important for bacterial attachment to host cell surface [9].

There are many different adhesion components existing on* H. pylori* to bind carbohydrates. The* sabA* has vital and important role in the primary colonization of* H. pylori. H. pylori sabA* proteins are also taking part in stable infections and development of chronic inflammation which directs to tissue damage [32].

*H. pylori sabA is a* diversity gene. The* sabA *gene has been associated with different stomach diseases such as 100% in gastric cancer, 86.7% in gastric ulcer, and 83.3% in gastritis and duodenal ulcer [33].

In conclusion, virulence factors of* H. pylori* gene sequences might differ markedly from other regions or other countries. These differences are detectable by PCR analysis and sequencing. These data suggest that* virulence factor* variants may present new markers for other factors involved in gastric carcinogenesis or probably influencing the result of* H. pylori* infection [34].

## Figures and Tables

**(a):** 

Primer	Primer sequence	Annealing Temperature (°C)	Primer Length	Product Size (bp)
AspA-F	ATGCGTATGGAGCATGATTTCATT	55.9	24	1401
AspA-R	TTTATGCTTTTTGAAAGCGTGAGGGCTT	62.2	28	

BabA-F	ATGAAAAAACACATCCTTTCATTA	52.5	24	2192
BabA-R	TTATTCAAATACACGCTATAGAGTCTT	57.4	27	

CagA-F	AATACACCAACGCCTCCAAG	57.3	20	1741
CagA-R	GCTGACAAAGGAGCACTTCC	59.4	20	

HomB-F	TACAGACGCTCAAGGCAATG	57.3	20	1005
HomB-R	TCTATGGGTAGGGCGTTTTG	57.3	20	

SabA-F	CTCTCTCTCGCTTGCGGTAT	59.4	20	187
SabA-R	TTGAATGCTTTGCCTCAATG	53.2	20	

VacA-F	ACAAACACACCGCAAAATCA	53.2	20	1624
VacA-F	AACGGCCACATTAGTGGAAG	57.3	20	

**(b):** 

Gender	Kind of disease	Number of patients	AspA+	BabA+	CagA+	HomB+	SabA+	VacA+	Mean Age
Male	NS	21	16	3	2	1	2	4	39.85±17.11
	GU	20	15	6	0	0	1	6	49.4±17.82
Female	NS	25	18	5	1	1	3	4	47.52±17.01
	GU	16	9	3	0	1	0	6	45.93±12.28

**(c):** 

	NS	GU	Total	*p* value
Number of patients	46	36	82	-
*AspA*	34 (73.91%)	25 (69.44%)	59 (71.95%)	<0.05
*BabA*	7 (15.21%)	9 (25%)	16 (19.51%)	ns
*CagA*	3 (6.52%)	0 (0%)	3 (3.65%)	ns
*HomB*	2 (4.34%)	1 (2.77%)	3 (3.65%)	ns
*SabA*	5 (10.86%)	1 (2.77%)	6 (7.31%)	ns
*VacA*	12 (26.08%)	8 (22.22%)	20 (24.39%)	ns

**(a):** 

Comparison	*p* value	95% CI
*CagA vs HomB*	ns	-0.18 to 0.13
*CagA vs SabA*	ns	-0.14 to 0.16
*CagA vs AspA*	<0.0001	0.50 to 0.81
*CagA vs BabA*	<0.05	0.002 to 0.31
*CagA vs VacA*	<0.05	0.002 to 0.314
*HomB vs SabA*	ns	-0.11 to 0.19
*HomB vs AspA*	<0.0001	0.52 to 0.83
*HomB vs BabA*	<0.05	0.02 to 0.33
*HomB vs VacA*	<0.05	0.02 to 0.33
*SabA vs AspA*	<0.0001	0.49 to 0.80
*SabA vs BabA*	ns	-0.009 to 0.302
*SabA vs VacA*	ns	-0.009 to 0.302
*AspA vs BabA*	<0.0001	-0.65 to -0.34
*AspA vs VacA*	<0.0001	-0.65 to -0.34
*BabA vs VacA*	ns	-0.15 to 0.15

**(b):** 

	NS	GU
*AspA*	<0.0001	<0.0001
*BabA*	<0.001	<0.001
*CagA*	<0.001	>0.05
*HomB*	<0.001	<0.001
*SabA*	<0.001	<0.001
*VacA*	<0.001	<0.001

## Data Availability

The data used to support the findings of this study are available from the corresponding author upon request.
